# High Stress Levels and Trust toward the Government Are Associated with More Positive Attitudes toward COVID-19 Vaccines among French Students: A Pilot Study

**DOI:** 10.3390/vaccines10091377

**Published:** 2022-08-24

**Authors:** Clémence Brun, Oulmann Zerhouni, Laurène Houtin, Alexis Akinyemi, Carla Aimé-Jubin, Jordane Boudesseul, Nicolas Pinsault

**Affiliations:** 1 AD-HOC Lab, 13 rue Hispano Suiza, 92270 Bois-Colombes, France; 2 TIMC-IMAG UMR CNRS 5525, ThEMAS Team, Université Grenoble Alpes, Domaine de la Merci, 5 Avenue du Grand Sablon, 38700 La Tronche, France; 3 Laboratoire Parisien de Psychologie Sociale, EA 4386 (équipe PS2C), 200 Avenue de la République, CEDEX, 92001 Nanterre, France; 4 Laboratoire Ethologie Cognition Développement, 200 Avenue de la République, 92000 Nanterre, France; 5 Instituto de Investigación Científica, Facultad de Psicología, Universidad de Lima, Av. Javier Prado Este 4600, Lima 33, Peru

**Keywords:** COVID-19, stress, vaccination, youth, trust

## Abstract

Mistrust in COVID-19 vaccines may hinder vaccination campaigns. We looked at cognitive determinants of vaccination intentions against COVID-19. We were interested in (i) the effects of stress and (ii) the effects of self-protection systems on attitudes and intentions to get COVID-19 vaccines. We conducted an online observational pilot study with 203 participants and used self-report questionnaires to assess perceived stress and vulnerability to disease, beliefs about a dangerous world, pandemic-related stressors, living conditions, attitudes and intentions toward the vaccines and trust in government management of the COVID-19 pandemic. The participants reporting high levels of trust in government and high levels of stress were more likely to have positive attitudes toward COVID-19 vaccines, although these two effects are at least partially independent of each other. We discuss how to improve the communication around COVID-19 vaccine policies.

## 1. Introduction

As of 3 February 2022, 370,572,213 confirmed cases of COVID-19 and 5,649,390 confirmed deaths were reported worldwide by the World Health Organization. Aside from alarming consequences for individual and collective health, researchers also report consequences on emotional and social functioning [1]. It appears that these consequences are particularly marked among young students. Indeed, recent studies conducted in Europe [2], UK [3], USA [4], Latin America [5] and Middle East [6] underlined the influence of the fear of COVID-19, stress and the anxiety related to depression and feeling of incompetence experienced by undergraduate university students. Moreover, the fear of COVID-19 could favor future career anxiety, creating a long-term negative effect on human psychology [7]. However, although several vaccines are available, part of the population remains reluctant to get vaccinated. Indeed, a survey among British adults showed that 64% of the participants were very enthusiastic to receive COVID-19 vaccines, while 27% were unsure and an alarming 9% were very unlikely to receive the vaccines [8]. Another study on French people showed that 35% of the sample were “hesitant” [9]. This goes hand in hand with a decrease in vaccination rates and an increase in refusal to receive vaccines over the past several years [10], particularly in France [11,12]. While vaccination against COVID-19 has been very successful in at-risk or elderly populations (e.g., over 84% in the US [13]), it is largely less prevalent in younger populations at a lower risk of developing severe forms of COVID-19 [14]. Perceived uncertainty around the health risks and benefits associated with a recently developed vaccine seems to be the main factor that hampers vaccination. For example, (i) some individuals are fearful and believe that vaccines pose risks of infection; (ii) some people underestimate the benefits of vaccines and/or the severity of some diseases; (iii) some individuals or populations have difficulty accessing vaccines [10,12]. While these factors are interesting and useful for understanding intentions to get vaccinated or not, they may not sufficiently consider individual differences in the processing of uncertainty about vaccination.

Hence, we investigated how stress responses induced by the pandemic situation (i.e., living conditions, socioeconomic variables) and individual self-regulatory capacities (i.e., perceived stress and protective systems) determine vaccination attitudes and intentions in a population of young people.

We looked at how protective systems (self-protection and disease avoidance systems) [15] modulate the stress response to vaccination intentions. Each system is underpinned by an excess of vigilance toward risk, tending toward self-protective responses even when potentially threatening cues are ambiguous. Each system is highly sensitive to context fluctuations and individual differences in vulnerability and is more likely to be engaged by those who feel chronically vulnerable to a specific threat. Each system is characterized by a coordinated sequence of mechanisms adapted to the perception of specific types of threat signals. These two systems have been evolutionarily developed to address two distinct categories of threats. The self-protection system is dedicated to the detection of emotional facial expressions, allowing the detection of the danger that others may represent. Because environmental threats are found not only in other individuals, but also in diseases and epidemics, humans have also developed a pathogen avoidance system, which has the function of guiding us away from elements that are likely to cause contamination.

While generic stress regulatory processes tend to direct humans toward handling stimuli as threats as the “default” mode [16], protective systems function to modulate this primary stress response by mitigating or augmenting it. Protective systems are, therefore, automatically engaged and then regulated in a more or less adapted manner. Koole [17] points out that there are important interindividual differences in psychophysiological traits related to self-regulation: these traits could determine the regulation of the engagement of protective systems.

We first hypothesized that distrust toward the government handling of the COVID-19 situation would lead to more negative attitudes toward the COVID-19 vaccination. Second, we hypothesized that the effect of distrust on attitudes would, in turn, be explained by higher levels of perceived stress, pathogen avoidance and self-protection on the one hand and by living conditions, environmental stressors and the COVID-19 burden on the other due to a greater propensity to perceive vaccines as threatening and a lower propensity to engage in prosocial behaviors in protecting others.

## 2. Materials and Methods

We determined the required sample size through an a priori power analysis using the G*Power software. We used a small-to-medium effect size, so our expected effect size was set at *f^2^* = 0.10. Because we ran two models, we used a Bonferroni correction on the alpha threshold (0.05/2 = 0.025). The analysis specifying a “linear multiple regression: fixed model, R2 deviation from 0” returned a minimum sample of 208 participants to ensure 90% power with the *p* set at 0.025 and six predictors. For the general population of 1,650,000 students in France and a sample of 203 participants, we had a margin of error of 7% at the 95% confidence level. We analyzed data from 203 French students (M*Age* = 19, SD*Age* = 2.52, women = 173). Data were collected from 1 December to 16 December 2020 through an online survey. This period follows the second lockdown in France to stem the resurgent coronavirus pandemic, with residents only allowed to leave their homes for food shopping, medical appointments, pressing family reasons and to commute to work when their jobs cannot be performed from home. Our study was composed of two sets of variables. On the one hand, we measured intentions to get vaccinated with RNA vaccines and traditional vaccines against COVID-19, the level of confidence in the French government regarding its management of the COVID-19 pandemic and the level of confidence in the epidemiological data issued by the government. On the other hand, we assessed the participants’ stress levels over the past month (with the Perceived Stress Scale (PSS) [18]), as well as the levels of trait protection and disease avoidance systems (Belief in a Dangerous World Scale and Perceived Vulnerability to Disease Scale [19,20]). Both the PVD and BDW were translated into French by the authors since there is no validated version in French. We also measured several indicators of the participants’ living conditions during periods of health restrictions and their concerns about the effects of the pandemic on their social, professional and personal lives [21]. The participants then completed a demographic questionnaire, questionnaires on psychological variables (randomized), followed by questionnaires regarding their living conditions and concerns (randomized) and outcomes related to their trust in government and vaccines.

### 2.1. Independent Variables

Trust toward government policies (TTG). The participants were asked to indicate whether, in general, they thought the French government was effective in managing the COVID-19 pandemic on a visual analog scale ranging from 0 (not at all) to 100 (definitely).

Perceived Stress Scale (PSS). The French version of the 10-item Perceived Stress Scale with a visual analog scale was used. The PSS assesses the degree to which an individual perceives life to be unpredictable and difficult for them to manage (ω = 0.88, α = 0.88, M = 59.2, SD = 19.1). The participants were asked to respond on a visual analog scale ranging from 0 (never) to 100 (often).

Belief in a Dangerous World (BDW). The Dangerous World Belief Scale measures the extent to which individuals tend to be chronically afraid for their safety and perceive the world to be full of dangers. The participants were asked to complete a five-point 12-item scale ranging from “strongly disagree” to “strongly agree” (ω = 0.84, α = 0.84, M = 3.22, SD = 0.68).

Perceived Vulnerability to Diseases (PVD). The 15-item Perceived Vulnerability to Disease Scale measures sensitivity to germ transmission (“germ aversion” dimension, eight items) and the feeling of being personally more sensitive to the risk of being infected (“pathogen sensibility” dimension, seven items). The participants answered on a seven-point scale ranging from “strongly disagree” to “strongly agree” (ω = 0.72, α = 0.75, M = 4.02, SD = 0.85).

Living conditions. We assessed data on lockdown conditions similar to the ones used by Flaudias et al. [22]. The questions focused on housing conditions and the number of people and children with whom individuals were confined (see Supplementary Materials for more details).

Pandemic-related stressors. We used the same scale as Flaudias et al. [22] to measure the worries related to the pandemic situation with a specific 11-item scale of stressors associated with specific lockdown concerns (e.g., “I am concerned about my finances”, “I am concerned about future employment opportunities”).

COVID-19 burden. The degree to which the participants were affected by COVID-19 was measured in three items (“Did you have a loved one infected/hospitalized/deceased with COVID-19) on a dichotomous scale (“yes” vs. “no”).

### 2.2. Dependent Variable

Attitudes toward the COVID-19 vaccines. The participants were asked two questions regarding their general attitude toward attenuated virus and RNA vaccines (“If an attenuated/RNA virus vaccine against COVID-19 were available now, do you think (i) it would be safe to administer it to the population, without the risk of unexpected side effects and (ii) it would be effective in fighting the COVID-19 pandemic?”) on a visual analog scale ranging from 0 (not at all) to 100 (definitely). Then, the participants were asked to indicate whether they would be willing to be vaccinated against COVID-19 if it were a messenger RNA vaccine or if it were an attenuated virus vaccine (i.e., a conventional vaccine, AV) on a visual analog scale ranging from 0 (not at all) to 100 (definitely). We computed the mean “attitudes toward vaccines” scores based on these six items, which showed extremely high reliability (ω = 0.93, α = 0.93, M = 31.9, SD = 24.2).

Statistical Analyses. We conducted two multiple linear mediation analyses with trust in the government policies as a predictor and the attitudes toward vaccines as an outcome. The first model included cognitive variables as mediators (PSS, BDW, PVD, or “Cog”) and the second model included environmental variables (i.e., living conditions, COVID-19 burden and stressors scores, or “Env”) as mediators. All the variables were centered. We expected an indirect effect of the trust in the government via PSS, BDW and PVD scores in the “Cog” model and mediation via living conditions, COVID-19 burden and stressor scores in the “Env” model. Age and sex were entered as covariates. Raw data are available at https://osf.io/se7gc (accessed on 21 August 2022).

## 3. Results

On average, the participants had relatively negative attitudes toward vaccines, i.e., below the mid-scale point (M = 32, SD = 24.2). Trust in the government management of the SARS-CoV-2 pandemic, data relative to the SARS-CoV-2 pandemic, regarding vaccine safety and efficacy were also relatively low, i.e., below the mid-scale point (respectively, M_management_ = 32.1, SD_management_ = 28.9, M_data_ = 32.8, SD_data_ = 26.6, M_safety_ = 32.8, SD_safety_ = 26.6, M_efficacy_ = 32.8, SD_efficacy_ = 26). All the descriptive statistics are available in Supplementary Materials. For both models, we report all the direct, indirect and total effects for both models and the significant effect for specific components. All the estimates can be found in Table 1.

Model-Cog: We found a total effect of the trust in the government as a predictor on the attitudes toward vaccines as an outcome, b = 0.69, z = 9.91, *p* < 0.001, such that a higher trust led to more positive attitudes toward vaccines. We found a significant indirect effect of the trust in the government on attitudes toward vaccines (TTG → PSS → vaccine attitudes), b = −0.05, z = −2.44, *p* = 0.01, a significant “a” component (TTG → PSS), b = −0.26, z = −4.35, *p* < 0.001, and a marginally significant “b” component (PSS → vaccine attitudes), b = 0.22, z = 2.94, *p* = 0.003. The lower the trust in the government, the higher the perceived stress, which turns to more positive attitudes toward vaccines. No significant mediation effect was found for the perceived vulnerability to disease and belief in a dangerous world scores (*p* > 0.14).

Model-Env: We found a total effect of the trust in the government as a predictor o f vaccine attitudes as an outcome, b = 0.70, z = 9.7, *p* < 0.001, such that a higher trust led to more positive attitudes toward vaccines. No significant mediation effect was found (*p* > 0.10).

## 4. Discussion

In an effort to understand vaccine hesitancy, we conducted an observational pilot study on 203 French participants. Not surprisingly, the factor that most robustly predicted both vaccination attitudes and intentions was confidence in the information provided by the government and its ability to manage the pandemic in general. France has opted for free access to vaccines and a specific communication campaign has been conducted to encourage young people to be vaccinated. However, this communication was mainly through the press and television, whereas social networks seem to have played a major role in the choice of vaccination, particularly among young people [23]. The lack of trust in government information and action appears to have a “direct” effect on attitudes toward vaccination, as well as an indirect effect via perceived stress. Thus, individuals who trust the government tend to have, not surprisingly, more positive attitudes toward vaccines, but also tend to feel less stress than those who do not trust the government. Thus, the decision to get vaccinated seems, at least in young people, to be quite far from reasoning processes that take into account the benefit-risk balance of being vaccinated against COVID-19, but rather to be the secondary consequences of self-regulatory (i.e., stress) and societal variables.

Individuals who are more stressed tend to have more positive attitudes toward vaccination. This is contrary to our hypotheses, where we assumed that individuals experiencing more stress would fall back on defensive, avoidance strategies, and therefore tend to have negative attitudes towards the vaccine. One possible explanation is that the COVID-19 pandemic is unique in that people’s stress and anxiety levels were not only related to getting sick or transmitting the disease to others, but also to the social and economic issues related to the measures taken by governments to contain the pandemic and stop the transmission of the disease between humans. We can also think that in the context of a pandemic, with the stress that it generates, the fact of learning that a new vaccine would allow us to get out of this pandemic situation would act as a sort of an anxiolytic. It would, therefore, be appropriate to examine the causal relationship between stress and vaccination, i.e., whether a high level of stress predicts the intention to be vaccinated, or whether, on the contrary, apart from medical considerations, if vaccination is used as a form of stress regulation.

*Limitations.* There are notable limitations in this study. In particular, as stated above, the environmental variables did not seem to have an influence on the participants’ intention to get vaccinated. This could be attributed to the fact that the environmental effect may be attributable to other variables that we did not consider in this study, which should be investigated in the future. It could also be due to the “global” character of the pandemic which could flatten the effect of the environmental variables on attitudes as everyone is, more or less, equally affected. This may also partly be explained by the fact that this pilot study was conducted exclusively on first-year psychology students, a kind of sample for which homogeneity is high [24]. As our pilot study was conducted on French participants, they may have a peculiar relationship with politics or current events, particularly in terms of distrust in the government. Although we have little reason to believe that our results are not generalizable to the population from which our sample was drawn, these results should be replicated on participants from different socioeconomic and ideological backgrounds to ensure the robustness and external validity of the results. Furthermore, our sample size (*n* = 203) as well as its composition limit the external and internal validity of our study. We emphasize that these results should be replicated on larger samples and in other cultures and socioprofessional categories, in particular because of the potential lack of statistical power that may limit the interpretation of certain analyses. An additional limitation is related to the overrepresentation of female participants in our sample (around 85%), even with taking into account our reference population, which may also undermine its representativeness of the university student population. Finally, because the study was conducted online, it is possible that the participants with greater ease and comfort in responding to an online study may have been overrepresented.

*Practical implications*. Thus, we have two different but related sources of impact on attitudes toward vaccination. The profile of individuals who would report having negative attitudes toward vaccination would include the following two characteristics: (i) low trust in the government and (ii) low stress. The latter may result from the fact that individuals may choose to cut themselves off from sources generating stress when it becomes too difficult to manage [18,25]. From a theoretical perspective, one might think that individuals choose to be vaccinated to reduce a level of stress that becomes difficult to bear. This suggests that measures to increase vaccination rates should be based on fear appeals, although this raises major ethical issues and may result in deleterious health effects that would outweigh those of vaccines. One solution would be to bolster individuals’ sense of self-efficacy while highlighting the real risks of not getting vaccinated [26]. Future research should focus on the interaction between the variables modulating the reception of information issued by public health authorities and the ability of individuals to make a truly informed and unbiased decision.

## 5. Conclusions

On the two sources of variation that explain the intention to get vaccinated, the stressful nature of the environment (i.e., an external variable) and the individual traits (i.e., an internal variable), we did not observe any mediating influence of the living and housing conditions, as well as the COVID-19 burden or environmental stressors on the attitude toward vaccination. It seems that it is not directly the living conditions and “objective” environmental elements that influence attitudes towards vaccines, but the potential impact they may have on individual variables that regulate behavior. Although we have not obtained clear results on this set of environmental variables, the way in which they interact and may have a causal impact on individual and cognitive variables remains an open question that can be answered by experimental (if possible) or longitudinal designs. Future research should focus more on understanding the mechanisms of the antecedents of stress response (i.e., the appraisal of the stimulus) in order to better design strategies on the stress response. We hope these preliminary results may be a gateway to more comprehensive and experimental studies of the factors affecting youths and the general population in general, attitudes and intentions to get vaccinated during this pandemic.

## Figures and Tables

**Table 1:** Mediation estimates for model-Cog and model-Env.

	Indirect and Total Effects								
					95% CI (a)				
Criterion	Type	Effect	Estimate	SE	Lower	Upper	β	z	*p*
Model-Cog	Indirect	Trust in the government ⇒ Perceived Stress Scale (PSS) ⇒ attitudes toward the SARS-CoV-2 vaccines	−0.05	0.02	−0.1	−0.01	−0.05	−2.44	0.01
		Trust in the government ⇒ Perceived Vulnerability to Diseases (PVD) ⇒ attitudes toward the SARS-CoV-2 vaccines	0.01	0.01	−0.01	0.017	0.01	0.66	0.5
Model-Cog		Trust in the government ⇒ Belief in a Dangerous World Scale (BDW) ⇒ attitudes toward the SARS-CoV-2 vaccines	0.01	0.02	−0.04	0.051	0.01	0.09	0.92
	Component	Trust in the government ⇒ Perceived Stress Scale (PSS)	−0.26	0.05	−0.38	−0.14	−0.31	−4.39	< 0.001
		Perceived Stress Scale (PSS) ⇒ attitudes toward the SARS-CoV-2 vaccines	0.22	0.07	0.07	0.37	0.17	2.94	0.003
		Trust in the government ⇒ Perceived Vulnerability to Diseases (PVD)	−0.01	0.01	−0.01	0.01	−0.09	−1.22	0.21
Model-Cog		Perceived Vulnerability to Diseases (PVD) ⇒ attitudes toward the SARS-CoV-2 vaccines	−1.36	1.7	−4.7	1.98	−0.04	−0.79	0.42
		Trust in the government ⇒ Belief in a Dangerous World Scale (BDW)	−0.01	0.01	−0.01	−0.01	−0.37	−5.49	<0.001
		Belief in a Dangerous World Scale (BDW) ⇒ attitudes toward the SARS-CoV-2 vaccines	−0.2	2.24	−4.6	4.19	−0.01	−0.09	0.92
	Direct	Trust in the government ⇒ attitudes toward the SARS-CoV-2 vaccines	0.69	0.07	0.55	0.83	0.65	9.91	<0.001
	Total	Trust in the government ⇒ attitudes toward the SARS-CoV-2 vaccines	0.64	0.06	0.51	0.76	0.6	10.11	<0.001
Model-Env	Indirect	Trust in the government ⇒ pandemic-related stressors ⇒ attitudes toward the SARS-CoV-2 vaccines	−0.03	0.02	−0.07	0.01	−0.03	−1.62	0.1
		Trust in the government ⇒ COVID-19 burden ⇒ attitudes toward the SARS-CoV-2 vaccines	−0.01	0.01	−0.01	0.01	−0.01	−0.52	0.59
Model-Env		Trust in the government ⇒ living conditions ⇒ attitudes toward the SARS-CoV-2 vaccines	0.01	0.01	−0.01	0.01	0.01	0.02	0.98
	Component	Trust in the government ⇒ pandemic-related stressors	0.01	0.01	0.01	0.01	0.25	3.09	0.002
		Pandemic-related stressors ⇒ attitudes toward the SARS-CoV-2 vaccines	−3.76	1.96	−7.62	0.08	−0.12	−1.91	0.05
		Trust in the government ⇒ COVID-19 burden	0.01	0.01	−0.01	0.01	0.08	1.04	0.29
		COVID-19 burden ⇒ attitudes toward the SARS-CoV-2 vaccines	−1.06	1.74	−4.48	2.35	−0.03	−0.6	0.54
		Trust in the government ⇒ living conditions	−1.15	0.87	−2.87	0.56	−0.1	−1.31	0.18
		Living conditions ⇒ attitudes toward the SARS-CoV-2 vaccines	0.01	0.01	−0.01	0.01	−0.01	−0.02	0.98
Model-Env	Direct	Trust in the government ⇒ attitudes toward the SARS-CoV-2 vaccines	0.7	0.07	0.56	0.84	0.65	9.7	<0.001
	Total	Trust in the government ⇒ attitudes toward the SARS-CoV-2 vaccines	0.66	0.07	0.52	0.8	0.61	9.4	<0.001

Note. Estimates of the total, direct, indirect and path “a” and “b” effects of mediation analyses for the Cog and Env models. Confidence intervals were computed using the standard (Delta) method. Betas are completely standardized effect sizes.

## Data Availability

All the raw data and materials (questionnaires) are available at https://osf.io/se7gc (accessed on 21 August 2022).

## Supplementary Materials

The following supporting information can be downloaded at: https://www.mdpi.com/article/10.3390/vaccines10091377/s1, Supplementary Section S1: Self-reported questionnaires, Supplementary Section S2: Sample Descriptive statistics.

## References

[1] Pfefferbaum B., North C.S. (2020). Mental health and the COVID-19 pandemic. N. Eng. J. Med..

[2] Elmer T., Mepham K., Stadtfeld C. (2020). Students under lockdown: Comparisons of students’ social networks and mental health before and during the COVID-19 crisis in Switzerland. PLoS ONE.

[3] Choi B., Jegatheeswaran L., Minocha A., Alhilani M., Nakhoul M., Mutengesa E. (2020). The impact of the COVID-19 pandemic on final year medical students in the United Kingdom: A national survey. BMC Med. Educ..

[4] Son C., Hegde S., Smith A., Wang X., Sasangohar F. (2020). Effects of COVID-19 on college students’ mental health in the United States: Interview survey study. J. Med. Internet Res..

[5] Rodríguez-Hidalgo A.J., Pantaleón Y., Dios I., Falla D. (2020). Fear of COVID-19, stress, and anxiety in university undergraduate students: A predictive model for depression. Front. Psychol..

[6] Zolotov Y., Reznik A., Bender S., Isralowitz R. (2022). COVID-19 fear, mental health, and substance use among Israeli university students. Int. J. Ment. Health Addict..

[7] Mahmud M.S., Talukder M.U., Rahman S.M. (2021). Does “Fear of COVID-19” trigger future career anxiety? An empirical investigation considering depression from COVID-19 as a mediator. Int. J. Soc. Psychiatry.

[8] Sherman S.M., Smith L.E., Sim J., Amlôt R., Cutts M., Dasch H., Sevdalis N. (2021). COVID-19 vaccination intention in the UK: Results from the COVID-19 vaccination acceptability study (CoVAccS), a nationally representative cross-sectional survey. Hum. Vaccin. Immunother..

[9] Detoc M., Bruel S., Frappe P., Tardy B., Botelho-Nevers E., Gagneux-Brunon A. (2020). Intention to participate in a COVID-19 vaccine clinical trial and to get vaccinated against COVID-19 in France during the pandemic. Vaccine.

[10] Kwok K.O., Li K.K., Wei W.I., Tang A., Wong S.Y.S., Lee S.S. (2021). Editor’s Choice: Influenza vaccine uptake, COVID-19 vaccination intention and vaccine hesitancy among nurses: A survey. Int. J. Nurs. Stud..

[11] Lazarus J.V., Ratzan S.C., Palayew A., Gostin L.O., Larson H.J., Rabin K., El-Mohandes A. (2021). A global survey of potential acceptance of a COVID-19 vaccine. Nat. Med..

[12] Liu J., Liao X., Qian S., Yuan J., Wang F., Liu Y., Zhang Z. (2020). Community transmission of severe acute respiratory syndrome Coronavirus 2, Shenzhen, China, 2020. Emerg. Infect. Dis..

[13] Diesel J., Sterrett N., Dasgupta S., Kriss J.L., Barry V., Esschert K.V., Barbour K.E. (2021). COVID-19 vaccination coverage among adults—United States, 14 December 2020–22 May 2021. Morb. Mortal. Wkly. Rep..

[14] Baack B.N., Abad N., Yankey D., Kahn K.E., Razzaghi H., Brookmeyer K., Singleton J.A. (2021). COVID-19 vaccination coverage and intent among adults aged 18–39 years—United States, March–May 2021. Morb. Mortal. Wkly. Rep..

[15] Neuberg S.L., Kenrick D.T., Schaller M. (2011). Human threat management systems: Self-protection and disease avoidance. Neurosci. Biobehav. Rev..

[16] Brosschot J.F., Verkuil B., Thayer J.F. (2016). The default response to uncertainty and the importance of perceived safety in anxiety and stress: An evolution-theoretical perspective. J. Anxiety Disord..

[17] Koole S.L. (2009). The psychology of emotion regulation: An integrative review. Cogn. Emot..

[18] Dupret E., Bocéréan C. (2013). La mesure du stress en milieu professionnel avec l’échelle de stress perçu (Perceived Stress Scale): Pertinence des versions en dix et quatre items. Psychol. Trav. Organ..

[19] Altemeyer B. (1988). Enemies of Freedom: Understanding Right-Wing Authoritarianism.

[20] Lazarus R.S. (1966). Psychological Stress and the Coping Process.

[21] Duncan L.A., Schaller M., Park J.H. (2009). Perceived vulnerability to disease: Development and validation of a 15-item self-report instrument. Pers. Individ. Dif..

[22] Flaudias V. (2021). The Early Impact of the COVID-19 Lockdown on Stress and Addictive Behaviours in an Alcohol-Consuming Student Population in France. Front. Psychiatry.

[23] Cascini F., Pantovic A., Al-Ajlouni Y.A., Failla G., Puleo V., Melnyk A., Ricciardi W. (2022). Social media and attitudes towards a COVID-19 vaccination: A systematic review of the literature. EClinicalMedicine.

[24] Henrich J., Heine S.J., Norenzayan A. (2010). Most people are not WEIRD. Nature.

[25] Cheetham M., Pedroni A.F., Antley A., Slater M., Jäncke L. (2009). Virtual milgram: Empathic concern or personal distress? Evidence from functional MRI and dispositional measures. Front. Hum. Neurosci..

[26] Tannenbaum M.B., Hepler J., Zimmerman R.S., Saul L., Jacobs S., Wilson K., Albarracín D. (2015). Appealing to fear: A meta-analysis of fear appeal effectiveness and theories. Psychol. Bull..

