# Supplementary material for: High Stress Levels and Trust toward the Government Are Associated with More Positive Attitudes toward COVID-19 Vaccines among French Students: A Pilot Study
Source: Vaccines (Basel). 2022 Aug 24;10(9):1377. doi: 10.3390/vaccines10091377 (PMC9505305; doi:10.3390/vaccines10091377)
Supplement: Supplementary file 1 [file vaccines-10-01377-s001.zip › vaccines-1857700-supplementary.pdf]

Supplementary Section S1—Self-Reported Questionnaires

Pandemic related stressors

Instructions: We're going to ask you a few things that can be stressful during Lockdown. Again, there is no right or wrong answer, we are only interested in your sincere answer. Participants had to answer on a scale from 1 (Strongly Agrees) to 7 (Strongly Disagrees)

1. My daily income

2. Being able to do my job well enough

3. Future employment prospects

4. Access to basic necessities such as food

Factor 1

Factor In review

5. Not being able to participate in group social activities

6. Dealing with the behavior of adults with whom I isolate myself

7. Dealing with the behavior of children with whom I isolate myself

8. The national economy

9. The risk that I or others I know will catch COVID-19

10. The risk of myself or others I know being hospitalized or dying because of

COVID-19

11. Adapting your work to digital platforms

12. Having to adapt one's social life to digital platforms

13. Being ashamed to act differently from others (e.g. at work, shopping)

Living conditions

Instructions: Indicate what best describes your current situation

• If you are locked down, how many other adults live together in the same place as

you?

• How many people live in the city where you live?

• What is the size of the place where you live during Lockdown (in m^2)?

• Does your living location have an exterior?

o Yes

o No

• If yes, do you have access to a:

o Balcony

o Garden

o Inner courtyard

If you are locked down, how many children under the age of 12 live together in the

same place as you?

• Do you know of anyone infected with Covid-19?

o Yes

o No

• Do you know anyone who's been hospitalized because of Covid-19?

o Yes

o No

• Do you know people who died because of Covid-19?

o Yes

o No

Belief in a dangerous world scale

Instructions : For each of the following statements, please indicate how much you agree with the statement : 1 = Strongly disagree, 2 = Disagree, 3 = Neither agree nor disagree, 4 = Agree, 5 = Strongly agree

• It seems that every year there are fewer and fewer truly respectable people, and more and

more persons with no morals at all who threaten everyone else.

• Although it may appear that things are constantly getting more dangerous and chaotic, it

really is not so. Every era has its problems, and a person's chances of living a safe,

untroubled life are better today than ever before.

• If our society keeps degenerating the way it has been lately, it's liable to collapse like a

rotten log and everything will be in chaos.

• Our society is not full of immoral and degenerate people who prey on decent people. News

reports of such cases are grossly exaggerating and misleading.

• The "end" is not near. People who think that earthquakes, wars and famines mean God

might be about to destroy the world are being foolish.

• There are many dangerous people in our society who will attack someone out of pure

meanness, for no reason at all.

• Despite what one hears about "crime in the street," there probably is not any more now than

there ever has been.

• Any day now, chaos and anarchy could erupt around us. All the signs are pointing to it.

• If a person takes a few sensible precautions, nothing bad will happen to him. We do not live

in a dangerous world.

• Every day, as our society becomes more lawless, a person's chances of being robbed,

assaulted, and even murdered go up and up.

• Things are getting so bad, even a decent law-abiding person who takes sensible precautions

can still become a victim of violence and crime.

• Our country is not falling apart or rotting from within.

Perceived Vulnerability to Disease scale

Instructions : For each of the following statements, please indicate how much you agree with the statement : 1 = Strongly disagree, 2 = Somewhat disagree, 3 = Slightly disagree, 4 = Neutral, 5 = Slightly agree, 6 = Somewhat agree, 7 = Strongly agree

• It really bothers me when people sneeze without covering their mouths.

• If an illness is 'going around', I will get it.

• I am comfortable sharing a water bottle with a friend.

• I do not like to write with a pencil someone else has obviously chewed on.

• My past experiences make me believe I am not likely to get sick even when my friends are

sick.

• I have a history of susceptibility to infectious disease.

• I prefer to wash my hands pretty soon after shaking someone's hand.

• In general, I am very susceptible to colds, flu and other infectious diseases.

• I dislike wearing used clothes because you do not know what the last person who wore it

was like.

• I am more likely than the people around me to catch an infectious disease.

• My hands do not feel dirty after touching money.

• I am unlikely to catch a cold, flu or other illness, even if it is 'going around'.

• It does not make me anxious to be around sick people.

• My immune system protects me from most illnesses that other people get.

• I avoid using public telephones because of the risk that I may catch something from the

previous user.

Supplementary Section S2—*Sample Descriptive statistics*

	**Overall (N=133)**
**Age**	
Mean (SD)	18.8 (1.8)
Range	13.0 - 29.0
**Gender-**	
Female	117 (88.0%)
Male	15 (11.3%)

	Overall (N=133)
Other	1 (0.8%)
**Year of study**	
1st year	130 (97.7%)
2nd year	1 (0.8%)
3rd year	1 (0.8%)
5th year	1 (0.8%)
**Job**	
Yes	25 (18.8%)
No	108 (81.2%)
**Scholarship**	
Yes	53 (39.8%)
No	80 (60.2%)
**Type of Housing**	
Personal Housing	8 (6.0%)
Roommate	4 (3.0%)
Student Residence	1 (0.8%)
Parent's house	120 (90.2%)
**Inner Courtyard (2)**	
Yes	24 (18.0%)
No	109 (82.0%)
**Balcony (2)**	
Yes	56 (42.1%)
No	77 (57.9%)
**Garden (2)**	
Yes	66 (49.6%)
No	67 (50.4%)
**Housing Area**	
Mean (SD)	207.7 (1259.3)
Range	17.2 - 14600.0
**City Population**	
Mean (SD)	187208.1 (536894.8)
Range	212.0 - 2148000.0
**How many adults do you live with?**	
Mean (SD)	2.2 (0.9)
Range	1.0 - 5.0

	Overall (N=133)
**How many children do you live with?**	
Mean (SD)	0.3 (0.7)
Range	0.0 - 3.0
**Living Conditions**	
Mean (SD)	72.3 (251.0)
Range	10.7 - 2925.0
**COVID-19 Burden**	
Mean (SD)	4.9 (0.9)
Range	3.0 - 6.0
**Pandemic-Related Stressors**	
Mean (SD)	20.8 (0.8)
Range	19.1 - 23.2
**Perceived Stress Scale (PSS)**	
Mean (SD)	59.2 (17.9)
Range	18.6 - 99.5
**Perceived Vulnerability to Diseases (PVD)**	
Mean (SD)	4.0 (0.8)
Range	1.9 - 5.9
**Belief in a Dangerous World Scale (BDW)**	
Mean (SD)	3.2 (0.6)
Range	1.8 - 4.8
**Trust in governement management of the** SARS-CoV-2 **pandemic**	
Mean (SD)	32.1 (22.8)
Range	0.0 - 92.0
**Trust in government data relative to the** SARS-CoV-2 **pandemic**	
Mean (SD)	38.2 (26.6)
Range	0.0 - 95.0
**Trust in governement regarding vaccine safety**	
Mean (SD)	32.8 (26.6)
Range	0.0 - 100.0
**Trust in governement regarding vaccine efficacy**	
Mean (SD)	32.8 (26.0)
Range	0.0 - 100.0
**Attitude toward** SARS-CoV-2 **Vaccine**	
Mean (SD)	32.0 (24.2)

	Overall (N=133)
Range	0.0 - 92.4

Note. Mean (standard deviation) and minimum and maximum value for each socio-demographic, cognitive and environmental variable and outcomes used in the analyses.
